# Secondary omental and pectoralis major double flap reconstruction following aggressive sternectomy for deep sternal wound infections after cardiac surgery

**DOI:** 10.1186/1749-8090-6-56

**Published:** 2011-04-18

**Authors:** Toshiro Kobayashi, Akihito Mikamo, Hiroshi Kurazumi, Ryo Suzuki, Bungo Shirasawa, Kimikazu Hamano

**Affiliations:** 1 Departments of Surgery and Clinical Science, Division of Cardiac Surgery, Yamaguchi University, Graduate School of Medicine, 1-1-1 Minami-Kogushi, Ube, Yamaguchi, 755-8505 Japan

## Abstract

**Background:**

Deep sternal wound infection after cardiac surgery carries high morbidity and mortality. Our strategy for deep sternal wound infection is aggressive strenal debridement followed by vacuum-assisted closure (VAC) therapy and omental-muscle flap reconstrucion. We describe this strategy and examine the outcome and long-term quality of life (QOL) it achieves.

**Methods:**

We retrospectively examined 16 patients treated for deep sternal wound infection between 2001 and 2007. The most recent nine patients were treated with total sternal resection followed by VAC therapy and secondary closure with omental-muscle flap reconstruction (recent group); whereas the former seven patients were treated with sternal preservation if possible, without VAC therapy, and four of these patients underwent primary closure (former group). We assessed long-term quality of life after DSWI by using the Short Form 36-Item Health Survey, Version 2 (SF36v2).

**Results:**

One patient died and four required further surgery for recurrence of deep sternal wound infection in the former group. The duration of treatment for deep sternal wound infection in the recent group was significantly shorter than that in previous group (63.4 ± 54.1 days vs. 120.0 ± 31.8 days, respectively; p = 0.039). Despite aggressive sternal resection, the QOL of patients treated for DSWI was only minimally compromised compared with age-, sex-, surgical procedures-matched patients without deep sternal wound infection.

**Conclusions:**

Aggressive sternal debridement followed by VAC therapy and secondary closure with an omental-muscle flap is effective for deep sternal wound infection. In this series, it resulted in a lower incidence of recurrent infection, shorter hospitalization, and it did not compromise long-term QOL greatly.

## Background

Deep sternal wound infection (DSWI) occurs less commonly after median sternotomy for cardiovascular surgery than after other major surgery. Its incidence is reported to be 1% to 5% and it is a life-threatening complication. The treatment of DSWI has evolved from closed mediastinal antibiotic irrigation to the primary use of a pectoralis muscle flap. Today, established treatment protocols include aggressive surgical debridement, delayed secondary closure, and plastic reconstruction with muscle and omental flaps [1-6]. Despite remarkable advances, mortality rate remains high, and this complication prolongs the hospital stay [7,8].

Vacuum-assisted closure (VAC) therapy was first established for the treatment of pressure ulcers and other chronic wounds [9,10]. Since then, the applications for VAC therapy have expanded widely and now include cardiac surgical infection [11]. The principle of this device is based on fixed negative pressure applied to the wound, resulting in effective wound drainage, decreased bacterial colonization and arteriolar dilatation, and the promotion of granulation.

Our former strategy for DSWI consisted of debridement of the infected sternum, although the sternum was preserved in about half the patients. Almost all patients underwent primary wound closure using omental flaps, but this resulted in high mortality and the frequent recurrence of infection. Our new strategy consists of aggressive sternal debriedment (total sternectomy) followed by VAC therapy and secondary wound closure with omental and bilateral pectralis major flap reconstruction. We analyzed the long-term outcome and quality of life (QOL) of patients treated with this strategy.

## Methods

Between January, 2001 and December, 2007, among 741 patients who underwent cardiac surgery through a median sternotomy, 16 (2.2%) acquired a DSWI involving the thoracic aortic graft and sternum. Wound classification was defined according to the Oakly classification [12]. All DSWIs were classified as EI Oakly classification type 2B wound infections associated with sternal osteomyelitis, with or without an infected retrosternal space. Superficial surgical site infections, sterilized sternal dehiscence, unknown results of bacterial culture from the wound, and endocarditis were excluded in this study. Data obtained from medical records included demographic information, primary operative procedures, the interval from surgery until the presentation of the wound infection, duration of VAC therapy, recurrence of wound infections, duration of treatment for the infection (calculated after the onset of infection to the day of healing according to surgeon's judgement), and pathogens isolated from wound bacterial cultures (Table 1, 2). Infection was diagnosed when purulent or serous exudate from the sternal wound was observed, with signs such as sternal pain, instability, rubor of the wound margins, wound dehiscence, and elevated inflammation parameters; after other causes of infectious origin were excluded. We followed up patients after discharge by telephone interview and by questioning the physicians in charge of the outpatient department at our institute.

**Table 1:** Patients' characteristics

Patient	Age (Years)	Gender	Risk factor	Primary procedure	Operation time
1	61	Male	DM	Cardiac trauma	180
2	70	Male	Smoking	CABG	153
3	77	Female	None	AVR	270
4	65	Male	None	CABG	420
5	77	Male	None	CABG	428
6	72	Male	DM	CABG	445
7	71	Male	DM	AVR	300
8	67	Male	HD	CABG	340
9	59	Male	None	Aorta	595
10	87	Female	None	AVR	504
11	70	Male	HD	CABG	325
12	74	Female	Steroid	Aorta	470
13	61	Female	Steroid	Aorta	568
14	76	Male	None	Aorta	683
15	79	Male	None	CABG	342
16	62	Male	None	Aorta	496

CABG: Coronary artery bypass grafting, AVR: Aortic valve replacement,

Aorta: Thoracic Aortic surgery,

DM: Diabetes mellitus, Smoking: Currently smoking, HD: Chronic renal failure requiring hemodialysis, Steroid: Steroidal usage.

**Table 2:** Characteristics of the deep sternal wound infections.

Patient	Age (Years)	Gender	Risk factor	Primary procedure	Operation time (minutes)	Duration for treatment (days)	Pathogens	Follow up Period (months)	Prognosis	Cause of death
1	61	Male	DM	Cardiac trauma	180	150	MRSA	76.4	Alive	-
2	70	Male	Smoking	CABG	153	135	MSSA	78	Alive	-
3	77	Female	None	AVR	270	120	MRSA	63.4	Alive	-
4	65	Male	None	CABG	420	60	MRSE	64.8	Death	Pneumonia
5	77	Male	None	CABG	428	131	MRSE	50	Alive	-
6	72	Male	DM	CABG	445	124	MRSA	55.3	Alive	-
7	71	Male	DM	AVR	300	Not available	MRSA	0.57	Death	DSWI
8	67	Male	HD	CABG	340	37	MRSA	54	Alive	-
9	59	Male	None	Aorta	595	40	Klebsiella	54.8	Alive	-
10	87	Female	None	AVR	504	48	MRSA	4.8	Death	Meningitis
11	70	Male	HD	CABG	325	66	MRSA	12	Death	Pneumonia
12	74	Female	Steroid	Aorta	470	34	MRSA	37.1	Alive	-
13	61	Female	Steroid	Aorta	568	51	Pseudomonaus	31.2	Alive	-
14	76	Male	None	Aorta	683	66	MSSA	28.2	Alive	-
15	79	Male	None	CABG	342	203	MRSA	12.6	Death	Pneumonia
16	62	Male	None	Aorta	496	26	MSSA	11	Alive	-

Total: total sternectomy, Partial: partial sternectomy, None: sternectomy was not performed.

OF: Omental flap, PF: Pectralis major flap, VAC: VAC therapy

Primary: primary wound closure, Secondary: secondary wound closure.

MRSA: Methicillin-resistant *Staphylococcus aureus*, MRSE: Methicillin-resistant *Staphylococcus epidermidis*. Klebsiella: *Klebsiella pneumoniae*, Pseudomonas: *Pseudomonas aeruginosa*.

DSWI: Deep sternal wound infection.

The "former" group consisted of seven patients treated between 2001 and 2003, with various methods. After opening the wound fully and removing all sternal wires, the extent of infection was assessed carefully by inspection to decide on the extent of resection. Three patients were treated by total sternectomy and primary wound closure with transposition of omental and/or pectoralis major flaps and occlusive continuous saline irrigation (Table 2); one patient was treated by partial sternectomy and primary wound closure with transposition of omental and pectoralis major flaps and occulusive continuous saline irrigation (patient 2); and three patients were treated by sternal preservation and delayed closure with omental or pectoralis major flaps (patients 3, 6 and 7). To prepare the omental flap, the lower edge of the midline wound incision was extended to the upper part of the abdomen. An omental pedicle was fully mobilized on the right gastroepiploic artery by dividing the branches up to the greater curvature of the stomach. The pedicle was brought up into the anterior mediastinum through the front of the liver and fixed to the upper part of the mediastinum. The bilateral pectoralis major muscle was fully mobilized following detachment of the costal insertion, without resecting the humeral insertion, then rotated and sutured together without tension on the midline in a ventral of the omentum flap [2-6]. On the cranial side, half of the clavicular attachment was divided, preserving continuity between the pectoralis-rect abdominis muscle.

The "recent" group consisted of nine patients treated since October, 2003, using our new method: total sternectomy after VAC therapy, followed by secondary closure with transposition of omental and pectoralis major flaps. We performed VAC therapy generally using commercial polyurethane foam sponge, sterilized in our hospital, which was cut and fitted into the mediastinal space. A 22 Fr. trocar catheter was inserted into the sponge and a single layer adherent dressing (Ioban™2 Special Incise Draip; 3M Healthcare; St. Paul, MN) was applied, then continuous suction between 100 and 120 mmHg was initiated via a wall suction system. Every 2 to 7 days, the sponge was changed under general anesthesia in the operating room. After removing the old dressing, the wound was inspected and a new sample was taken for bacterial cultures. Necrotic tissue was removed and the wound was irrigated with copious amounts of warm saline. Timing for the termination of VAC therapy and delayed closure were decided by the following criteria: no pyrexia, decline of serological inflammation parameters, at least two negative bacterial cultures, and resolution of the local infection. We performed secondary definitive closure with omental flap transposition to fill the mediastinal space and reconstruction with bilateral pectoralis major flaps covering the anterior chest wall, as described above. The subcutaneous tissue and skin were closed and a silastic drain (BLAKE Drain; Ethicon, Inc., a Johnson & Johnson Company; Somerville, NJ) was left in the subcutaneous and pectoralis pockets and under the omental flap. All drainage tubes were connected to reservoirs (J-VAC Reservoires. Ethicon, Inc., a Johnson & Johnson Company; Somerville, NJ) and continuous suction was initiated. Postoperatively, patients received 2-4 weeks of intravenous antibiotics after the specific antibiogram, followed by at least 2 weeks of oral antibiotics.

To evaluate the long-term quality of life after DSWI treatment with our method, especially in relation to the problems associated with total sternal resection, we assessed the postoperative QOL of the seven patients who underwent total sternectomy, by using the Short Form 36-Item Health Survey, Version 2 (SF36v2) and compared the findings with age-, sex-, surgical procedure- and follow-up period-matched patients who had undergone cardiovascular surgery without a postoperative wound infection in our institute [13-15]. This consisted of 36 short questions mirroring health and QOL, based on eight aspects: physical functioning (PF, 10 items); role physical (RP, 4); body pain (BP, 2); general health (GH, 5); vitality (VT, 4); social functioning (SF, 2); role emotional (RE, 3), and mental health (MH, 5). The norm-based scoring algorithms introduced for all eight scales employ a linear score transformation, which scores scales with a mean of 50 and a standard deviation of 10 in the 2002 Japanese general population. The differences in scale scores clearly reflects the impact of the disease or treatment: any score lower than 50 falls below the general population mean, and each point represents 1/10th of a standard deviation.

This study was approved by the Medical Ethics Committee of Yamaguchi University School of Medicine, and informed consent was obtained from all the patients enrolled.

### Statistical Analysis

All values are expressed as means ± standard deviation. Comparisons between the two groups were established with unpaired *t *tests for continuous variables and with the χ^2 ^tests and Fisher's exact test for discrete variables. Differences were considered significant when the *p-*value was less than 0.05. All analyses were performed with the StatView 4.1 statistical software package (Abacus Concepts, Berkeley, California).

## Results

The mean follow-up periods were 64.7 ± 11.1 months for the former group and 21.0 ± 12.9 months for the recent group. The preoperative characteristics, including age, gender, risk factors for wound infections, primary operative procedures, and operation times, are listed in Table 1 and the characteristics of DSWI in each patient were listed in Table 2. The duration between the primary procedure and the clinical manifestation of infection were 13.4 ± 4.7 days (range, 7 to 17 days) in former group and 18.9 ± 18.7 days (range, 8 to 62 days) in recent group, respectively. The duration of VAC therapy (recent group) was 22.6 ± 11.7 days (range, 7 to 42 days). The mean duration of treatment for DSWI was shorter in the recent group than in the former group (63.4 ± 54.1 days vs.120.0 ± 31.8 days, respectively; p = 0.039). Four of the former group patients suffered recurrence of the infection, necessitating further surgery; namely, total sternectomy with primary wound closure in two and secondary wound closure without sternal resection in two. One of the latter patients (patient 7) died of sepsis caused by the DSWI, 17 days after the reoperation. Two of the recent group patients died of pneumonia and one of meningitis.

**Figure 1:**
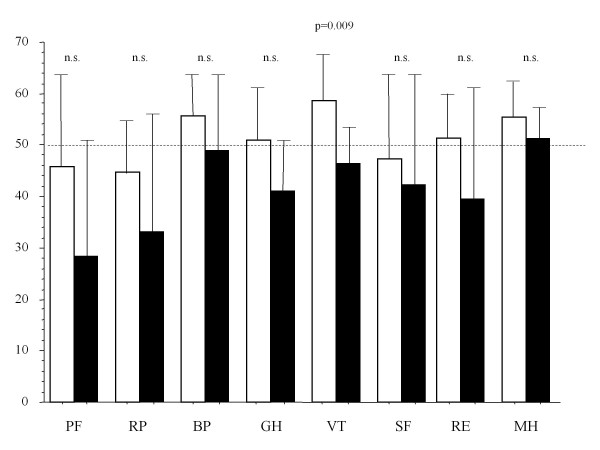
**QOL of patients treated with total sternectomy**. Age-, gender-, surgical procedures-, and follow-up period-matched comparison of the aspects assessed with the Short Form 36-Item Health Survey, Version 2 (SF36v2) in the patients who underwent total sternectomy (black bars) compared with patients who underwent cardiovascular surgery without DSWI (white bars). Score scales have a mean of 50 and a standard deviation of 10 in the 2002 Japanese general population.

Figure 1 shows the results of SF36v2 in the patients who underwent total sternectomy (patients 5, 8, 9, 12, 13, 14, and 16 in Table 1) and the patients without a sternal infection, at the time of assessment, a mean 47.3 ± 27.3 months after discharge. Patients who underwent total sternectomy had significantly lower scores in only 'vitality', when compared with age-, sex-, surgical procedures- and follow-up period-matched patients who underwent cardiovascular surgery without DSWI (46.4 ± 2.6 vs. 58.7 ± 3.2, respectively; p = 0.009). The other scores did not differ significantly between the two groups.

## Discussion

Sternal osteomyelitis is a serious postoperative complication with a mortality rate of about 30% [16]. Its management requires repeat operations and there are many risks, including life-threatening sepsis leading to multiple organ failure. Conventional treatment consists of massive sternal debridement and prolonged antibiotic therapy, which has many side effects and creates multi-resistant bacterias. Moreover, it requires long-term hospitalization.

Vacuum-assisted closure (VAC) therapy is based on fixed negative-pressure applied to the wound, resulting in drainage of the wound fluid, decreased bacterial colonization, arteriolar dilatation, and granulation. Previous studies have reported that VAC resulted in a low rate of recurrent infections and shorter hospitalization [17]. Accordingly, we observed superior effectiveness with VAC therapy and delayed wound closure with the transposition of omental and bilateral pectoralis major flaps. Before we decided to use VAC therapy, we examined what other methods were used, including massive sternal debridement, and primary or delayed closure with the transposition of omental and/or bilateral pectoralis major flaps. In these patients, closed drainage tubes were inserted around the mediastinal and subcutaneous space, with continuous or daily irrigation until the bacterial culture was negative. These treatments have some drawbacks such as bleeding and delayed early postoperative rehabilitation because of the multiple tubes in place for irrigation and suction. These disadvantages impaired the long-term treatment of infection, resulting in a high rate of recurrence (4 of 7 patients: 57.1%). Many authors have reported a high incidence of recurrence after primary closure, despite the use of various flaps [18-20]. Conversely, VAC therapy resulted in effective wound drainage and the promotion of granulation. In this series, there was no bleeding during VAC therapy with only a single tube for generating negative pressure, so the patients could eat and walk with ease. Thus, there was no recurrence of infection and treatment times were shorter.

In Japan, there is no commercial VAC therapy system, so we developed one using commercial polyurethane foam sponge, sterilized in our hospital. After being fashioned to the specific wound geometry, the sponge is placed into the wound. A single, straight 22 French trocar catheter is inserted directly into the sponge, and the wound site and anterior chest are covered with an adhesive drape, thereby covering an open wound into a controlled closed wound. The trocar catheter was connected to wall suction via a long tube, and negative pressure between 100 and 120 mmHg was generated. Patients treated with VAC therapy can ambulate by clamping the trocar catheter and disconnecting the tube from wall suction.

Some reports emphasize that sternal preservation and rewiring can be done by using VAC therapy, resulting in good quality of life, and that transposed omentum or muscle flaps are unnecessary afterwards [21-25]. The extent and degree of infection determines whether the sternum can be preserved. A high rate recurrence of infection when the sternum was preserved despite VAC therapy has been reported. To reduce the risk of recurrence of the infection, our strategy for complete treatment of wound infections consists of aggressive debridement of the infectious sternum (total sternectomy) and drainage with VAC therapy, followed by secondary definitive closure, with the transposition of omentum to fill the entire defect and bilateral pectoralis major flaps to reconstruct the anterior chest wall. Recurrence of infection is associated with high mortality, so we routinely transposed the omentum in addition to aggressive debridement following VAC therapy for several weeks. The omental flap is the best selection for preventing recurrence of an infection because of its abundant lymphoid tissues and ability to regenerate blood vessels [4-6]. After sterility of the mediastinal space has been achieved by VAC therapy, harvesting the omentum would not induce the intraperitoneal spread of infection. The omental flap can fill the whole space, but we used bilateral pectoralis major flaps to build the anterior chest wall, rather than to fill the dead space. Thus, we did not have to resect the humeral insertion, avoiding limitation of shoulder motion, muscle weakness, pain, and paresthesia, and securing blood supply to this muscle flap, even though the internal thoracic artery, a source of blood supply to the pectoralis major muscle, had to be separated from the chest wall when an arterial graft was needed in coronary artery bypass surgery.

The optimal timing of secondary closure following VAC therapy is not established. Ronny et al reported the effectiveness of the C-reactive protein level in VAC therapy [22]. We took bacterial cultures from the mediastinal space at the time of sponge exchange and when two negative cultures were confirmed, secondary closure was done. Although this needs clarification, we have not observed recurrence of infection after treatment with our new strategy. In comparison with age-, sex-, primary surgical procedure-, and follow-up period-matched patients without DSWI, the QOL of patients treated with total sternectomy was satisfactory in all regards except for 'vitality'. Immer et al reported that patients treated with sternal excision and reconstruction with a musculocutaneous flap showed a significant limitation of QOL, as assessed by SF36 in 6 of 8 aspects, although this was probably related to their general health in addition to the sternal wound healing problem [24]. Our study confirms that our recent strategy for DSWI, including aggressive sternal resection does not impair QOL. The reason for the lower 'vitality' of patients after total sternectomy was the muscle weakness of the lower extremities caused by long-term hospitalization, rather than to the wound causing pain and respiratory difficulties.

In conclusion, our strategy for DSWI, consisting of aggressive sternal debridement followed by VAC therapy and secondary closure with the transposition of omental and bilateral pectoralis major flaps, controls wound infection and reduces hospitalization. The long-term QOL achieved is comparable with that of patients without DSWI.

## Competing interests

The authors declare that they have no competing interests.

## Authors' contributions

TK developed study protocol, obtained data, analyzed data and wrote manuscript. AK developed the study protocol and provided critical revision of the manuscript. HK and RS and BS provided critical revision of the manuscript. KH conceived the study, developed study protocol, analyzed data and provided critical revision of the manuscript. All authors read and approved the final manuscript.
